# Bleeding complications from the direct oral anticoagulants

**DOI:** 10.1186/s12878-015-0039-z

**Published:** 2015-12-24

**Authors:** Michelle Sholzberg, Katerina Pavenski, Nadine Shehata, Christine Cserti-Gazdewich, Yulia Lin

**Affiliations:** Division of Hematology, Department of Medicine and Department of Laboratory Medicine and Pathobiology, St. Michael’s Hospital, University of Toronto, 30 Bond Street, Room 2-007G Core Lab, Carter Wing, Toronto, ON M5B-1 W8 Canada; Division of Hematology, Department of Medicine and Department of Laboratory Medicine and Pathobiology St. Michael’s Hospital, University of Toronto, Toronto, Ontario Canada; Departments of Medicine and Laboratory Medicine and Pathobiology, Mount Sinai Hospital, University of Toronto, Toronto, ON Canada; Department of Laboratory Medicine and Pathobiology, University Health Network, University of Toronto, Toronto, ON Canada; Department of Clinical Pathology, Sunnybrook Health Sciences Centre; and Department of Laboratory Medicine and Pathobiology, University of Toronto, Toronto, ON Canada

**Keywords:** Anticoagulants, Blood transfusion, Dabigatran, Hemorrhage, Rivaroxaban

## Abstract

**Background:**

Direct oral anticoagulants (DOACs) are now standard of care for the management of thromboembolic risk. A prevalent issue of concern is how to manage direct oral anticoagulant (DOAC)-associated bleeding for which there is no specific antidote available for clinical use. We conducted a retrospective case series to describe the Toronto, Canada multicenter experience with bleeding from dabigatran or rivaroxaban.

**Methods:**

Retrospective chart review of DOAC bleeding necessitating referral to hematology and/or transfusion medicine services at five large University of Toronto affiliated academic hospitals from January 2011 to December 2013.

**Results:**

Twenty-six patients with DOAC bleeding were reviewed; 42 % bleeds intracranial and 50 %, gastrointestinal. All patients had at least one risk factor associated with DOAC bleeding reported in previous studies. Inconsistent bleed management strategies were evident. Median length of hospital stay was 11 days (1–90). Five thromboembolic events occurred after transfusion based-hemostatic therapy and there were six deaths.

**Conclusions:**

Management of DOAC bleeding is variable. Clinical trial data regarding DOAC reversal is needed to facilitate optimization and standardization of bleeding treatment algorithms.

## Background

Vitamin K antagonists have long been the mainstay of prophylactic or therapeutic anticoagulation for thromboembolism. The cumbersome disadvantages of warfarin from both the patient and physician perspective have led to the development, and now standard use, of direct oral anticoagulants (DOACs) that do not require laboratory monitoring and have fewer food and drug interactions.

**Table 1:** Risk factors associated with direct oral anticoagulant bleeding

Clinical Variable	Number of patients
>80 years of age	12
Weight < 63 kg	4
Severe (<30 ml/min) or moderate (30–50 ml/min) impairment in creatinine clearance	9
Diabetes mellitus	5
Concomitant aspirin	5
Concomitant NSAID^a^	5
Concomitant strong P-gp^b^ inhibitors	3
Higher than recommended dose of dabigatran	1

^a^non-steroidal anti-inflammatory drug

^b^p-glycoprotein

**Table 2:** Hemostatic therapy according to bleed site

Intracranial Hemorrhage (ICH) Events (N = 11) 2 combined ICH and GIB	Gastrointestinal Bleed (GIB) Events (N = 12) 2 combined ICH and GIB
6 (54 %) received aPCC^a^	1 (8 %) received aPCC
0 (0 %) received PCC^b^	2 (17 %) received PCC
	2 (17 %) received both aPCC and PCC
7 (64 %) received hemostatic support of any kind	9 (75 %) received hemostatic support of any kind
4 (36 %) did not receive hemostatic therapy	3 (25 %) did not receive hemostatic therapy

^a^activated prothrombin complex concentrate

^b^prothrombin complex concentrate

**Fig. 1:**
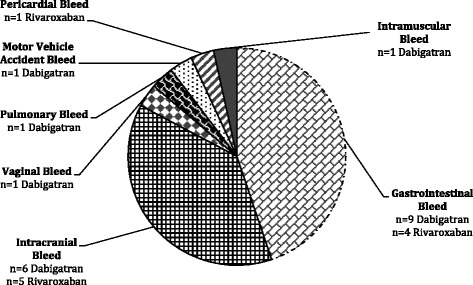
Bleed sites (*N* = 27 bleeding episodes in 26 patients)

Large clinical trials comparing the DOACs to vitamin K antagonists have demonstrated similar efficacy in the management and prevention of thromboembolism and similar or reduced major bleeding rates [1–3]. As indications for DOACs expand, an issue of concern is how to manage real-world DOAC-associated bleeding for which no antidote is currently available. Guidelines and reviews have extrapolated bleeding management principles from results of animal and human volunteer studies with laboratory, not clinical, parameters as primary outcomes [4–7]. Since no evidence-based, standard therapeutic algorithm for DOAC bleeding is available, the primary objective of our study was to determine how patients are currently being managed in this setting. We focused on the experience with hemorrhage from dabigatran, a direct thrombin inhibitor, and rivaroxaban, a direct factor Xa inhibitor, as apixaban, a direct factor Xa inhibitor, was not yet approved for use in Canada.

## Methods

We conducted a retrospective chart review of DOAC bleeding necessitating referral to hematology and/or transfusion medicine services at five large University of Toronto affiliated academic hospitals (St. Michael’s Hospital, Toronto General Hospital, Toronto Western Hospital, Sunnybrook Health Sciences Centre, Mount Sinai Hospital) from January 2011 to December 2013. Patients were included if they were: over the age of 18 years, documented to have a DOAC associated hemorrhage and identified to hematology and/or transfusion medicine services.

The following data were abstracted from medical records: age and sex; body weight; DOAC type; indication for DOAC; duration of time on DOAC therapy until bleeding event (days); concomitant medication use; initial blood work (including complete blood cell count, activated partial thromboplastin time (aPTT), prothrombin time (PT), fibrinogen (Claus method), liver enzymes (aspartate aminotransferase [AST], alanine transaminase [ALT], alkaline phosphatase [ALP]), albumin, bilirubin, estimated creatinine clearance (Cockcroft-Gault formula); description of bleeding episode (site, date/time documented, red blood cell (RBC) transfusion, severity of bleed – major or minor). Major bleeding was defined according to the International Society on Thrombosis and Haemostasis (ISTH)’s recommendations [8] as either involvement of a critical organ, fall in haemoglobin of more than 20 g/L or requirement of greater than two RBC transfusions. Of note, aforementioned data points included those known to be associated with increased risk of DOAC bleeding.

Additional data collected included: management of bleeding (DOAC held, site compression, surgical management, fluids/adequate urine output, charcoal, haemodialysis, transfusion [activated prothrombin complex concentrate (aPCC), prothrombin complex concentrate (PCC), activated recombinant factor VII, frozen plasma, platelets, cryoprecipitate, fibrinogen concentrate] and non-transfusion based [tranexamic acid, desmopressin, vitamin K] hemostatic support; coagulation based test results post-transfusion therapy; bleeding outcome (decrease, increase, no change, cessation); venous or arterial thromboembolic (TE) event (with supportive imaging results and/or blood work); length of hospital stay; and hospital discharge status (alive, dead).

Data were analyzed using descriptive statistics (mean, median, range and standard deviation) and inferential statistics (confidence interval). All analyses were performed using SAS statistical software, version 9.2 (SAS Institute Inc). Approval to perform this study and to report the results was obtained from St Michael’s Hospital Research Ethics Board, University Health Network Research Ethics Board associated with Toronto General Hospital and Toronto Western Hospital, the Human Research Protections Program associated with Sunnybrook Health Sciences Centre, and Mount Sinai Hospital Research Ethics Board. The aforementioned list of research ethics committees approved this study and granted access to medical records and databases at their respective hospital sites. Approval to publicize the data set was not obtained by the hospital Research Ethics Boards. Hospitals are required to protect the privacy of citizens whose information they collect. The hospitals strive to comply with the Personal Health Information Protection Act (PHIPA). Therefore data supporting the study findings are unavailable.

## Results

Twenty-seven bleeding events were captured upon retrospective review; one patient had two events hence a total of 26 patients were reviewed. Nine bleeding events occurred with rivaroxaban while 18 occurred with dabigatran. All except four patients were over the age of 70 years with a median age of 78 years (range 52–91 years). Approximately 69 % (18/26) of patients were male. Three individuals were underweight (less than 60 kilograms) while the median weight was 78.3 (range 50–150) kilograms. The median time taking the DOAC prior to bleeding was 120 days (range 5–810). The indications for DOAC therapy included the following: atrial fibrillation (*n* = 24), deep vein thrombosis (*n* = 1), and two patients (7 %) treated for an off-label indication (one for cancer associated pulmonary embolism and the other for prophylaxis for an automated implantable cardioverter-defibrillator. Of the nine rivaroxaban associated bleeds, five occurred at a dosage of 20 mg daily and four at 15 mg daily. Of the 18 dabigatran bleeds, eight occurred at a dosage of 150 mg twice per day and ten at 110 mg twice per day. The median number of concomitant medications was 7 (range 1–16). Five bleeding events occurred while the patient was taking concomitant aspirin therapy, five events with non-steroidal anti-inflammatory drugs (NSAIDs) and three occurred with concomitant P-glycoprotein (P-gp) inhibitors.

Eighty-nine percent of the bleeding events were classified as a major hemorrhage with 50 % requiring RBC transfusion. Of those who were transfused with RBCs, a median of 2.5 units (range 1–9) was required. Half of the dabigatran patients were transfused with RBCs (median 3 units, range 1–3) compared to 44 % treated with rivaroxaban (median 2 units, range 1–4).

Eleven (42 %) bleeding events were intracranial (ICH) and 13 (50 %) were gastrointestinal (GI) in origin. Of note, two of these events were combined ICH and GI hemorrhages. Of the 13 GI hemorrhages, nine were associated with dabigatran use and four with rivaroxaban. There were 11 ICH events, six occurred in dabigatran users and five in rivaroxaban users. There was no statistically significant association between dabigatran versus rivaroxaban use and type of hemorrhage using a two-tailed Fischer’s exact test (*p* = 0.68). More patients with GI bleeds received RBC transfusion (62 %), as compared to ICH (27 %).

The remaining four bleeds involved the following sites: vaginal, pulmonary, subcutaneous or musculoskeletal with some occurring in combination. One of these events was associated with a motor vehicle collision (Fig. 1).

Data were reviewed for risk factors (found in previous observational studies) associated with DOAC bleeding. In this study, all bleeding events occurred in the context of at least one previously identified associating factor and 63 % occurred with more than one. Specifically, 50 % of subjects were above 80 years of age and 33 % of cases occurred with severe (<30 ml/min) or moderate (30–50 ml/min) impairment in creatinine clearance at time of bleeding. Five subjects had comorbid diabetes mellitus, five were on concomitant aspirin and another 19 % were taking a NSAID (Table 1). Of the 26 patients, 14 were on a reduced dose of dabigatran (110 mg) or rivaroxaban (15 mg). All of those patients were either of older age or had impaired renal function. Fifty percent of those on standard dose DOAC were older or had abnormal kidney function.

Of the 18 dabigatran related bleed events (in 17 patients), five (28 %) received aPCC alone, two (11 %) received aPCC and PCC, 13 (72 %) received at least one hemostatic support of any kind and five (28 %) did not receive any hemostatic therapy. Of the nine rivaroxaban related bleeding events, six (67 %) received at least one hemostatic support (two (22 %) received aPCC, two (22 %) received PCC) and three (33 %) did not receive any hemostatic therapy.

APCC tended to be administered to a larger number of patients with ICH - six (54 %) - compared with isolated GI hemorrhage - one (8 %). However, a similar proportion of individuals received hemostatic therapy of any kind (63 % for ICH and 69 % for GI bleed) (Table 2).

Hemostatic response to aPCC and/or PCC seemed to differ according to the DOAC. Of the nine total patients that received aPCC, three (all dabigatran related) had resolution of bleeding within 12 to 24 h of administration. Of the five total patients that received PCC, one bleed (rivaroxaban related) had resolution of bleeding at 24 h and there was no abnormal intra-operative bleeding in another rivaroxaban related case. CBC, PT and aPTT were the most commonly ordered initial laboratory tests. However, the frequency of repeated testing was highly variable. Concise summarization of laboratory data was not possible. Furthermore, we cannot comment on the pattern of coagulation study normalization due to inconsistencies within the dataset.

There were five TE events in subjects who received transfusion based hemostatic therapy (i.e. aPCC, PCC, FP and/or platelets). All of the events were arterial in nature involving either myocardial infarction or bowel ischemia. A single arterial TE event occurred within 24 h of hemostatic transfusion (aPCC). There were no TE events in patients who did not receive transfusion based hemostatic therapy.

The median length of hospital stay was 11 days (range 1–90). There were six deaths (four dabigatran and two rivaroxaban) (23 % of cases). The cause of death was ICH in five patients and one death occurred secondary to multi-organ failure and myocardial infarction. The proportion of ICH resulting in death was 45 %.

## Discussion

We found, in this case series, that the management of DOAC bleeding is highly variable. A large proportion of patients in this study required prolonged hospitalization and experienced TE complications. Additionally, a substantial proportion of patients died. Of interest, 33 % of the patients in this case series would not have qualified for enrolment in the studies on the respective drugs.

This study is limited by the lack of control and denominator data, however, it highlights the importance of ‘risk’ factors for major DOAC related bleeding namely, advanced age, renal impairment, diabetes mellitus and concomitant treatment with aspirin and NSAIDs [9–12]. Despite reassuring recent evidence from a meta-analysis reviewing the bleeding rates in the elderly from published randomized controlled trials comparing DOACs with standard anticoagulant therapy, this study suggests that careful patient selection for treatment with DOACs remains paramount [12]. Interestingly, data from this case series is in contrast to the recently published data on rivaroxaban bleeding from the Dresden registry [13]. Most notably, this case series confirms that a high proportion of patients who experience anticoagulant associated ICH die [14].

## Conclusion

In summary, this case series shows that there is considerable variation in the treatment used to control DOAC associated bleeding. This study also highlights the importance of proper patient selection for DOAC therapy. In conclusion, management of DOAC bleeding needs to be optimized and standardized once clinical trial data regarding reversal becomes available.

## Abbreviations

ALP: Alkaline phosphatase
ALT: Alanine transaminase
AST: Aspartate aminotransferase
aPCC: Activated prothrombin complex concentrate
aPTT: Activated partial thromboplastin time
DOAC: Direct oral anticoagulants
GI: Gastrointestinal
ICH: Intracranial hemorrhage
ISTH: International society of thrombosis and haemostasis
NSAID: Non-steroidal anti-inflammatory drug
PCC: Prothrombin complex concentrate
P-gp: P-glycoprotein
PT: Prothrombin time
RBC: Red blood cell
TE: Thromboembolic
